# Flip Lists in Elective Hip and Knee Arthroplasty: A Retrospective Cohort Study on Efficiency and Patient Outcomes

**DOI:** 10.7759/cureus.98397

**Published:** 2025-12-03

**Authors:** Nimra Akram, Sharo Naqar, Ayman Ahmed, Irrum Afzal, Arif Moghal, Sarkhell Radha

**Affiliations:** 1 Trauma and Orthopaedics, South West London Elective Orthopaedic Centre, Epsom, GBR; 2 Trauma and Orthopaedics, Croydon University Hospital, London, GBR; 3 Orthopaedics, Imperial College London, London, GBR; 4 Orthopaedics, South West London Elective Orthopaedic Centre, Epsom, GBR; 5 Anaesthesia, South West London Elective Orthopaedic Centre, Epsom, GBR

**Keywords:** long-term clinical outcomes, orthopaedic surgery, primary total knee arthroplasty, theatre efficiency, total hip arthroplasty (tha)

## Abstract

Aims

This study evaluates the impact of implementing a 'flip list' approach in elective orthopaedic surgery on surgical efficiency, patient satisfaction, and resource utilisation.

Methods

A retrospective cohort study was conducted at South West London Elective Orthopaedic Centre, Epsom General Hospital, London, using prospectively collected data from a high-volume elective orthopaedic centre between August 2019 and April 2024. A total of 373 surgical lists (116 flip, 257 non-flip) involving 1,079 procedures were analysed. A total of 859 arthroplasties were analysed (523 total hip arthroplasty (THA), 326 total knee arthroplasty (TKA), and 10 unicompartmental knee arthroplasty (UKA) cases). Data included patient demographics, intraoperative variables, and post-operative outcomes. Key outcomes included theatre efficiency (procedure count, duration, touch time), postoperative outcomes (length of stay, complication/readmission rates), patient-reported outcome measures (PROMs)/patient-reported experience measures (PREMs), and income generated. Statistical analysis was performed using t-tests, Chi-square, and Fisher’s exact tests (p < 0.05 considered significant).

Results

Flip lists facilitated significantly more procedures per list (3.17 vs. 2.77; *p* < 0.001) with reduced operative duration (68 vs. 91 minutes) and shorter hospital stays (1.83 vs. 2.24 days; *p *< 0.001). No significant differences were observed in complication rates, readmissions, mortality, or patient-reported outcomes over a two-year follow-up. Economic analysis showed comparable income per case, but greater total revenue per list due to increased volume.

Conclusion

Flip lists enhance theatre efficiency and throughput in elective arthroplasty without compromising safety or patient satisfaction. The model aligns with Getting It Right First Time (GIRFT) principles and offers a scalable, high-impact solution for emerging high-volume elective orthopaedic centres aiming to meet rising demands whilst maintaining quality of care.

## Introduction

The National Health Service (NHS) continues to experience unprecedented challenges, with surgical waiting lists recently reaching historic highs [1]. Studies indicate that longer surgical waiting times lead to rapid declines in patients’ preoperative physical and mental health, increasing the risk of complications and prolonged recovery, which in turn places significant strain on secondary care and increases demands on primary and social care services postoperatively [2,3]. Due to medical advancements and increasing life expectancy, the demand for hip and knee arthroplasty is ever-increasing [4,5,6]. Addressing this demand and tackling the waiting-list backlog is imperative to enhance efficiency and mitigate the adverse effects on patients awaiting these life-changing surgical procedures.

The Getting It Right First Time (GIRFT) initiative, first introduced in 2016, aims to optimise surgical list efficiency and elevate standards of patient care in the NHS [7,8]. Healthcare trusts and surgical hubs have adopted various strategies to align with elective targets, particularly within the HVLC (high-volume, low-complexity) programme [8]. Hip and knee arthroplasties are key procedures within the HVLC framework, given their high demand and suitability for streamlined, standardised care pathways [9,10].

A unique approach at a high-volume elective orthopaedic centre is the implementation of flip lists. These lists, also known as overlapping lists, involve a single consultant surgeon moving across operating theatres to minimise turnover time and maximise touch time (the duration of direct interaction between the clinician and patient) [11]. This approach, supported by dedicated theatre teams, centres the consultant surgeon on critical elements of each procedure before safely moving on to the next case in a separate operating theatre. Whilst flip lists have shown promising results in other surgical specialities in the UK, their general application to orthopaedic surgery is not well known [11].

The efficiency and productivity of flip lists have been a subject of debate when compared to two independent lists managed by separate surgeons in their own theatres. In addition, questions have been raised regarding patient safety and inferior outcomes with flip/overlapping lists [11,12,13]. To address these concerns, this study evaluates the impact of the flip-list approach on surgical efficiency, patient outcomes, and hospital resource utilisation in elective orthopaedic surgery. The primary aim is to compare patient outcomes between flip and non-flip lists (with the same operating surgeon), focusing on patient-reported outcome measures (PROMs), patient-reported experience measures (PREMs), readmission rates, complication rates, patient satisfaction at six weeks, and 90-day mortality.

To our knowledge, this is the first study to offer an in-depth insight into the implementation of flip lists at a high-volume elective arthroplasty centre. Unlike previous studies that primarily focus on theatre efficiency and scheduling, this study addresses theatre efficiency, cost analysis, and patient outcomes within elective orthopaedics. By providing a comprehensive evaluation, it contributes evidence on how flip lists can safely enhance productivity in high-demand surgical environments.

## Materials and methods

Study design

This study is a retrospective cohort analysis of prospectively collected data, evaluating the impact of the flip list approach. It was conducted at the South West London Elective Orthopaedic Centre, Epsom General Hospital, London. The study compares data from patients managed under the flip list approach with those managed on traditional non-flip lists between August 2019 and April 2024 under the care of a single experienced high-volume orthopaedic surgeon. The flip list approach involved alternating the preparation and surgical execution of cases between two operating rooms, allowing the surgeon to move between rooms to minimise downtime. This process aimed to optimise the use of surgical teams and reduce non-operative time. The traditional non-flip lists followed a standard single-room procedure list, where each surgery was completed before the next was prepared. Each operating room contained its own scrub staff, anaesthetic team, and surgical assistant.

Setting and participants

The study was conducted at a high-volume elective orthopaedic centre and well-established surgical hub. We included patients undergoing total hip arthroplasty (THA) and total knee arthroplasty (TKA) during the study period. A total of 373 surgical lists were analysed, comprising 116 flip lists and 257 non-flip lists. The patients’ demographic characteristics, including age, sex, comorbidities, and preoperative health status, were recorded for both groups. A further analysis focusing solely on THAs and knee arthroplasties was also conducted.

Inclusion Criteria

The patient selection criteria were all patients undergoing primary elective THA, TKA and unicompartmental knee arthroplasty (UKA) from a single surgeon between the period of August 2019 to April 2024.

Exclusion Criteria

Patients undergoing revision surgery, when analysing solely arthroplasty procedures, were excluded.

Data collection

Data were extracted from the hospital’s electronic health records and operative databases. The following variables were collected: 1) patient demographics (age, sex, and American Society of Anesthesiologists (ASA) grade); 2) intraoperative variables (type of procedure, duration of surgery (from incision to wound closure), and touch time (from anaesthetic start time to wound closure)); 3) preoperative outcomes (Oxford Hip Score (OHS), Oxford Knee Score (OKS), and EQ-5D score); 4) postoperative outcomes (length of hospital stay (days), complications within 30 days post-surgery (orthopaedic and non-orthopaedic complications), 30-day readmission rates, patient satisfaction to the service and outcome, OHSs or OKSs and EQ-5D scores at six weeks, six months, one year, and two years); and 5) economic variables (income per case and overall income per list).

Only patients with complete data for each variable were included in the study. Any patients with missing data were therefore excluded to allow for accurate and complete comparison. 

Statistical analysis

All statistical analyses were performed using IBM SPSS Statistics for Windows, Version 29.0.2.0 (20) (released 2022, IBM Corp., Armonk, NY). Continuous variables were expressed as means with standard deviations, and categorical variables were presented as proportions.

Independent t-tests and Mann-Whitney U tests were used to compare continuous variables between the flip list and non-flip list groups. Chi-squared tests were employed to assess differences in categorical variables. Fisher’s exact tests were also applied for analysing overruns and readmissions between the two groups.

A p-value of <0.05 was considered statistically significant for all comparisons.

Ethical considerations

The study was approved by the local hospital following institutional review. All data were anonymised prior to analysis. There was no additional patient contact, and as such, this study was performed as a service evaluation without the need for formal ethical approval. The study was conducted in accordance with the Declaration of Helsinki and the guidelines for good clinical practice. Explicit clearance for the use of validated scoring systems (OHS, OKS, and EQ-5D) was obtained from our centre’s research department. The institution’s research department holds clearance from the original developers for the use of validated scoring systems in clinical research.

## Results

Overview of the surgical list

A total of 373 elective orthopaedic surgical lists were analysed: 116 flip lists and 257 non-flip lists. Flip lists included 368 surgeries (average 3.17 procedures per list), compared to 711 surgeries in the non-flip group (average 2.77 procedures per list).

 The demographic characteristics between the two groups were similar; however, patients on flip lists were on average 1.83 years older (67.16 vs. 65.33) (Table 1).

**Table 1:** Comparison of patient demographics between flip and non-flip lists Age was compared between groups using an independent-samples t-test. Sex distribution (male %) and ASA grade were compared using chi-square tests (Cramer’s V reported for effect size in ASA). All p-values are two-sided, with significance set at p<0.05. ASA: American Society of Anesthesiologists

Patients' demographic characteristics	Flip lists (n = 116)	Non-flip lists (n = 257)	Test statistics	p-value
Age (years, mean±SD)	67.16 ± 11.15	65.33 ± 12.19	t = 2.310	0.021
Sex (% male)	39.5% (145/367)	41.6% (296/711)	χ² = 0.367	0.544
ASA grade (distribution)	2 (64.67%)	2 (59.21%)	χ² = 7.667, Cramers V = 0.084	0.053

Procedure breakdown

Flip lists included 211 THA, 124 TKAs, and six UKA cases. Non-flip lists included 312 THAs, 202 TKAs, and four UKAs. This equated to a significantly higher average number of arthroplasties per flip list (2.94 vs 2.02, p < 0.05), suggesting that flip lists can facilitate a greater number of arthroplasties in the same timeframe as non-flip lists.

**Figure 1:**
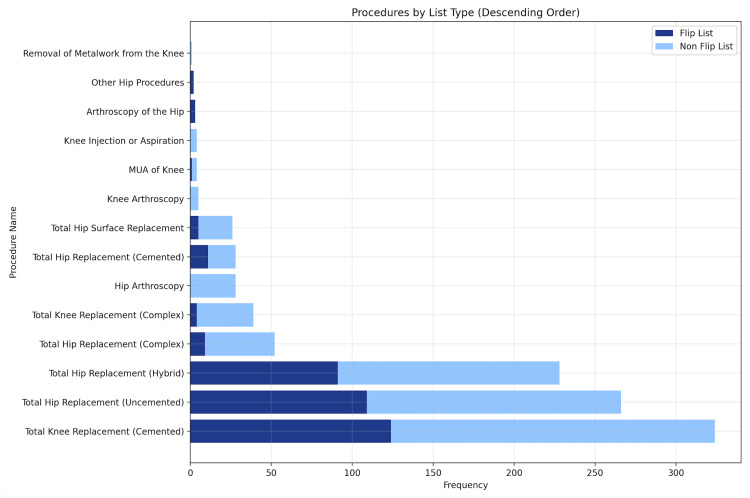
Number and diversity of procedures performed in flip (n = 368) and non-flip lists (n = 711)

The flip lists included a wide variety of procedures such as THAs, hip resurfacing, UKA, TKA revisions, and other hip procedures (e.g, injections, aspirations, and excision of ossification/bursa) (Figure 1).

Intraoperative efficiency

Surgical metrics favoured flip lists. The average number of procedures performed per list was higher in the flip list group compared to the non-flip list group (3.17 vs. 2.77, p < 0.001). The average length of surgery for primary arthroplasty procedures was significantly shorter in the flip list group (68 minutes vs. 91 minutes, p < 0.001), as was the total touch time in the operating room for arthroplasty (99 minutes vs. 115 minutes, p < 0.001). The average anaesthetic time for procedures on flip lists was 13 minutes, compared to 14 minutes on non-flip lists. This difference was statistically significant (p < 0.001) (Table 2). Considering these results, the flip list model enabled more procedures per list and per day, demonstrating its value in streamlining surgical throughput. 

**Table 2:** Comparison of surgical efficiency between flip and non-flip lists All surgical efficiency variables (procedures per list, arthroplasty surgery duration, arthroplasty touch time, and arthroplasty anaesthetic time) were compared between flip and non-flip lists using independent-samples t-tests. All p-values are two-sided, with significance set at p < 0.05.

Surgical variable	Flip lists	Non-flip lists	Test statistic	p-value
All procedures per list (mean ± SD)	3.17 ± 0.70	2.77 ± 0.99	t = 4.527	<0.001
Arthroplasty surgery duration (minutes)	67.49 ± 17.53	90.50 ± 51.19	t = −9.43	<0.0001
Arthroplasty touch time (minutes)	99.06 ± 20.82	114.78 ± 26.52	t = −9.70	<0.0001
Arthroplasty anaesthetic time (minutes)	12.72 ± 7.16	14.13 ± 7.65	t = −2.73	0.0064 < 0.05

Postoperative outcomes

The length of hospital stay was significantly reduced in patients managed with the flip list approach compared to those on non-flip lists (1.83 days vs. 2.24 days, p < 0.001) (Table 3). Day-case surgeries were more common in the flip list group (23.1% vs. 16.5%). Complication rates were slightly lower in the flip group (4.89% vs. 5.77%), although the difference was not statistically significant (Table 4). The 30-day readmission rate was also lower in the non-flip list group, although this difference was not statistically significant (2.45% vs. 1.55%, p = 0.34). There were no 90-day mortalities in either group.

**Table 3:** Comparison of orthopaedic and non-orthopaedic complications in flip and non-flip lists. Complication rates were compared between flip and non-flip lists. Fisher’s exact test was used for outcomes with low event counts (≤5 events in either group), while chi-square tests were applied to complications with higher frequencies (e.g., urinary tract infection). Results are reported as event counts (n, %), test statistic (odds ratio where calculable), and two-sided p-values. Statistical significance was set at p < 0.05.

Orthopaedic complications	Flip list (n = 368)	Non-flip list (n = 711)	Test statistic (odds ratio)	p-value
Dislocation	0 (0%)	0 (0%)	—	—
Deep-vein thrombosis (DVT)	0 (0%)	2 (0.28%)	—	0.5551
Pulmonary embolism	0 (0%)	1 (0.14%)	—	1.0000
Joint infection	1 (0.27%)	1 (0.14%)	OR=0.77	1.0000
Superficial infection	0 (0%)	1 (0.14%)	—	1.0000
Nerve palsy	1 (0.27%)	2 (0.28%)	OR=0.58	0.5599
Fracture	2 (0.54%)	2 (0.28%)	OR=0.58	0.5599
Summary (orthopaedic)	1.09%	1.27%	—	1.0000
Non-orthopaedic				
Myocardial infarction	0 (0%)	0 (0%)	—	—
Pneumonia	1 (0.27%)	4 (0.56%)	OR=0.32	0.4332
Stroke	0 (0%)	1 (0.14%)	—	1.0000
Urinary tract infection (UTI)	13 (3.53%)	27 (3.80%)	OR=0.80	0.4255
Summary (non-orthopaedic)	3.80%	4.50%	—	0.6371
Overall complications	4.89%	5.77%	—	0.6720

**Table 4:** Comparison of patient demographics, intraoperative and postoperative variables for flip lists and non-flip lists. Analysis was also completed for theatre utilisation and income generated for flip and non-flip lists. Continuous normally distributed variables (age, number of procedures per list, surgical duration, anaesthetic time, touch time, and income) were analysed using independent-samples t-tests (two-sided). Non-normally distributed continuous variables (number of procedures per day, theatre utilisation measures, and length of stay) were compared using the Mann–Whitney U test. Categorical variables (gender, ASA grade, laterality, and procedure type) were assessed using chi-square tests. Due to low event counts, binary outcomes, including overruns, readmissions, complications, and 90-day mortality, were analysed with Fisher’s exact test. Statistical significance was defined as p < 0.05.

Variables	Flip	Non-flip	Test statistic	p-value
Age (mean ± SD, yrs)	67.16 ± 11.15	65.33 ± 12.19	t = 2.31	0.021
Gender (male, %)	39.51%	41.63%	χ² = 0.37	0.544
ASA grade (median)	2	2	χ² = 7.67	0.053
Number of procedures/day (mean ± SD)	6.46 ± 1.39	2.77 ± 0.99	U = 12.5	<0.001
Number of procedures/list (mean ± SD)	3.17 ± 0.70	2.77 ± 0.99	t = 4.53	<0.001
Procedure type			χ² = 79.8	0.004
Laterality (left, %)	46.7%	46.1%	χ² = 0.24	0.889
Length of surgery (min)	68 ± 18	92 ± 51	t = –9.43	<0.001
Anaesthetic time (min)	12.7 ± 7.2	14.1 ± 7.7	t = –2.73	0.006
Touch time (min)	99 ± 21	115 ± 27	t = –9.70	<0.001
Theatre utilisation – capped (%)	57.3	67.0	U = 5554.5	0.012
Theatre utilisation – uncapped (%)	57.7	67.8	U = 2327.5	<0.001
Theatre utilisation – raw (%)	76.6	83.8	U = 7.1	<0.001
Length of stay (days)	1.83 ± 2.61	2.24 ± 2.23	t = –2.70	0.007
Overruns (%)	4.31% (5/116)	8.95% (23/257)	Fisher’s exact OR = 0.46	0.139
Readmissions (%)	2.45% (9/368)	1.55% (11/711)	Fisher’s exact OR = 1.60	0.343
Orthopaedic complications (%)	1.09%	1.27%	Fisher’s exact OR = 0.86	1.000
Non-orthopaedic complications (%)	3.80%	4.50%	Fisher’s exact OR = 0.84	0.637
90-day mortality (%)	0	0	—	—
Income generated (mean, £)	7751.2	7761.8	t = –0.10	0.921

When analysing complication rates, flip lists had four orthopaedic complications from 368 procedures, with non-flip lists having nine orthopaedic complications in 711 procedures. Notably, the flip list group had fewer postoperative infections and thromboembolic events.

Patient-reported outcomes and satisfaction

OHS and OKS showed no significant difference between groups at preoperative, six weeks, one-year, and two-year. PREMs were equivalent at all time points (Table 5).

**Table 5:** Comparison of patient demographics, intraoperative and postoperative variables specifically for hip and knee arthroplasty cases undertaken in flip lists versus non flip lists. Age, length of surgery, anaesthetic time, touch time, and length of stay were compared using independent-samples t-tests (two-sided). Gender, ASA grade, and laterality were compared using chi-square tests. The number of arthroplasties per list and per day was compared using Poisson/Chi-square rate tests. All analyses were two-sided with a significance threshold of p < 0.05. ASA: American Society of Anesthesiologists

Variables	Flip	Non-flip	Test statistic	p-value
Age (mean ± SD, yrs)	67.58 ± 11.13	67.06 ± 12.15	t = 0.70	0.484
Gender (male, %)	39.3%	40.0%	χ² = 0.02	0.902
ASA grade (median)	2	2	χ² = 5.66	0.129
Number of arthroplasties/list (mean ± SD)	2.94 ± 0.70	2.02 ± 0.99	χ² = 7.88	<0.001
Number of arthroplasties/day (mean ± SD)	5.98 ± 1.39	2.02 ± 0.99	Z = 4.70	<0.001
Laterality (left, %)	46.9%	47.9%	χ² = 0.04	0.838
Length of surgery (min, mean ± SD)	68 ± 18	91 ± 51	t = –9.43	<0.001
Anaesthetic time (min, mean ± SD)	13 ± 7.2	14 ± 7.7	t = –2.73	0.006
Touch time (min, mean ± SD)	99 ± 21	115 ± 27	t = –9.70	<0.001
Length of stay (days, mean ± SD)	1.81 ± 2.61	2.13 ± 2.23	t = –2.15	0.032

Overall, these findings suggest that there are no notable differences in clinical outcomes or patient satisfaction for THAs and TKAs performed using flip versus non-flip lists (Table 6).

**Table 6:** Patient-reported outcome measures and patient-reported experience measures in terms of validated Oxford Hip Scores (OHS) and Oxford Knee Scores (OKS) and outcome satisfaction for both flip and non-flip list patient cohorts Continuous patient-reported outcome measures (PROMs) (Oxford Hip Score, Oxford Knee Score) were compared between flip and non-flip lists using independent-samples t-tests. Changes from baseline to follow-up were analysed with t-tests on change scores. Patient-reported satisfaction (percentage scores) was compared between groups using independent t-tests. All p-values are two-sided, with significance set at p < 0.0.

Variable	Flip (mean ± SD)	Non-flip (mean ± SD)	Test statistic	p-value
Preoerative OHS	16.52 ± 8.9	16.11 ± 9.1	t = 0.74	0.461
1-year OHS	41.57 ± 8.3	41.05 ± 8.6	t = 0.85	0.398
2-year OHS	43.47 ± 7.9	42.21 ± 8.0	t = 1.08	0.280
Change pre-op → 1 year	25.05 ± 8.7	24.94 ± 8.8	t = 0.58	0.565
Change pre-op → 2 years	26.95 ± 8.4	26.10 ± 8.5	t = 1.82	0.068
Oxford Knee Score (OKS)	Flip (mean ± SD)	Non-flip (mean ± SD)	Test statistic	p-value
Preoperative OKS	15.87 ± 7.5	15.93 ± 7.6	t = –0.96	0.337
1-year OKS	35.51 ± 8.2	36.02 ± 8.5	t = –0.76	0.451
2-year OKS	38.79 ± 8.0	39.20 ± 8.2	t = –1.08	0.280
Change pre-op → 1 year	19.64 ± 6.2	20.09 ± 6.1	t = –2.72	0.007
Change pre-op → 2 years	22.92 ± 6.9	23.27 ± 6.8	t = –1.82	0.068
THA – outcome satisfaction (%)	Flip (mean ± SD)	Non-flip (mean ± SD)	Test statistic	p-value
6 weeks	90.61 ± 6.8	90.53 ± 7.0	t = 0.04	0.968
6 months	90.87 ± 6.6	90.83 ± 6.8	t = 0.35	0.729
1 year	91.37 ± 6.5	91.49 ± 6.7	t = –0.15	0.881
2 years	91.88 ± 6.4	91.64 ± 6.6	t = 0.09	0.931
TKA – outcome satisfaction (%)	Flip (mean ± SD)	Non-flip (mean ± SD)	Test statistic	p-value
6 weeks	86.92 ± 7.4	86.97 ± 7.6	t = –0.35	0.729
6 months	87.30 ± 7.1	87.27 ± 7.3	t = 0.91	0.361
1 year	88.06 ± 7.0	88.18 ± 7.2	t = –0.60	0.552
2 years	88.76 ± 6.9	88.47 ± 7.0	t = 0.28	0.781

Theatre efficiency and economic impact

Theatre overruns occurred in 4.31% of cases on flip lists, where the parallel list typically finished earlier. By contrast, non-flip lists experienced overruns in 8.95% of cases. Income per case was similar between the two models, but flip lists generated higher total revenue due to increased daily case volume.

## Discussion

This study provides the first UK-based evaluation of the flip list model in elective orthopaedic surgery, focusing on its impact on efficiency, safety, and patient-reported outcomes. The findings demonstrate that flip list significantly enhances surgical throughput and reduces intraoperative and perioperative inefficiencies without compromising patient safety, satisfaction or outcomes. The flip list model enabled significantly more procedures per list and per day, supported by shorter operative and anaesthetic times, and a higher proportion of day-case surgeries. These gains were achieved without increased complications or readmission rates. Patient-reported outcomes and satisfaction, measured through validated PROMs and PREMs, were equivalent between flip and non-flip groups at all time points. These findings suggest that the flip list approach is not only efficient but also clinically safe and acceptable to patients.

While the flip list approach has been explored in other surgical specialities such as cardiac and general surgery, its value in orthopaedics remains under-reported. Most research on surgical efficiency has focused on reducing theatre turnover times or streamlining workflows. Studies in general surgery and cardiac surgery have demonstrated that overlapping surgeries or staggered operating rooms can improve output without compromising patient safety [11,12]. Our findings suggest that with effective workforce management and surgical expertise, flip lists can enhance the safety, efficiency, and overall effectiveness of care for patients undergoing elective joint replacements [9].

Previous research by Ravi et al. raised concerns about overlapping surgeries, suggesting they may increase complication risks, particularly in hip procedures involving fractures and arthritis [9]. Ravi et al. found that overlapping surgeries were associated with higher complication rates, especially when the overlap duration increased. This highlights the importance of patient consent and careful selection for such procedures. However, our findings challenge these concerns. Our data showed no significant differences in patient outcomes or patient satisfaction between flip and non-flip lists. In fact, patients undergoing THAs and TKAs on flip lists experienced fewer complications, suggesting that the structured and organised nature of flip lists can mitigate the risks associated with overlapping surgeries. These results indicate that with meticulous planning and coordination, the flip list approach enhances efficiency without increasing risks, providing a safe alternative to traditional surgical models.

Implementing flip lists does present several challenges, particularly for the anaesthetic team. Frail and complex patients often require longer preparation times for invasive monitoring techniques, such as central venous catheters (CVCs) and arterial lines, which are crucial for patient safety [14]. In addition, the growing use of regional anaesthesia and nerve blocks has extended preparation times. However, incorporating complex patients into a flip list, where they are prepared while the surgeon is operating on another patient, allows these challenges to be managed more efficiently.

Increased anaesthetic preparation time in overlapping surgical lists (flip lists) offers valuable educational opportunities for resident doctors. The extended preparation period allows anaesthetists to engage in teaching activities, thereby enhancing foundational knowledge and clinical skills without compromising operative efficiency. Similarly, during the wound closure phase, the consultant surgeon transitions to the next operating room to initiate the subsequent case. This handover of closure tasks to resident doctors provides a focused environment for developing essential wound closure skills. The reduced time pressure compared to single-room workflows harbours a more thorough learning experience and skill development, ultimately contributing to improved clinical outcomes and operational efficiency [15]. Despite concerns that underutilised theatre space might reduce departmental productivity, our findings suggest that well-organised and executed flip lists offer a more efficient alternative.

Beyond clinical workflow, flip lists have significant workforce and educational implications. While one theatre is in use, anaesthetic teams and surgical trainees can focus on preparation, teaching, and closure in a less pressured environment. This distributed responsibility model not only supports staff development and morale but also enhances educational opportunities without affecting overall productivity.

The improved outcomes observed in our study can be attributed to several factors inherent to the flip list approach. Firstly, by allowing for the preparation of one patient while surgery is ongoing in an adjacent theatre, flip lists minimise idle time and ensure that theatre resources are used more efficiently. This translates into shorter overall operating room times, as our data show a reduction in theatre overruns from 4.31% in non-flip lists to just 8.95% in flip lists. This reduction in theatre overruns is critical, as it allows for more predictable surgical schedules, reducing staff fatigue and improving workflow consistency.

The ability to manage complex patients more efficiently is a key strength of the flip list model. As our data suggest, anaesthetic teams can prepare high-risk patients with greater care and attention in a flip list, without the pressure of immediate surgical demands. The structured nature of the flip list encourages a more deliberate and methodical approach to patient preparation, allowing for optimal intraoperative management, particularly in patients requiring invasive monitoring or advanced regional anaesthesia techniques.

The implementation of flip lists closely aligns with the GIRFT and NHS directive, which aims to optimise theatre time, reduce delays, and increase case volume within existing resources [15]. Our flip list approach helped in maximising surgical capacity, reducing delays, and maintaining high clinical standards. These outcomes are particularly relevant as the NHS seeks innovative, sustainable methods to address elective backlogs.

In addition to improving operational efficiency, the flip list approach has had a positive impact on staff morale at the high-volume elective orthopaedic centre. The structured and collaborative nature of flip lists reduces the burden on individual team members, addressing concerns of burnout and improving staff retention. The prioritisation of cases and the smooth transitions between theatres have also reduced the overall waiting time for patients, leading to higher patient satisfaction. By 2022, three years after implementation, growing staff experience and workflow familiarity enabled the inclusion of complex arthroplasties and revision procedures in flip lists through strategic case planning.

Limitations

This was a retrospective, single-surgeon study, introducing a possibility of selection bias and limiting generalisability. Although patient characteristics were broadly similar between groups, there may have been unmeasured differences in complexity. Additionally, theatre staff experience and anaesthetic support may have influenced results.

As surgical demand continues to rise, especially for procedures like THA and TKA, the need for innovative models like flip lists becomes increasingly important. Investigating the model's impact on trainee development, long-term outcomes, and cost-effectiveness is important for the future, considering the increasing demands.

## Conclusions

In a high-volume elective orthopaedic centre, the flip-list model increased procedures per list and shortened operative and inpatient timelines without compromising safety, readmissions, or patient-reported outcomes. These gains translated to greater revenue per list via higher throughput rather than higher income per case. When accompanied by governance, clear consent, parallel anaesthetic preparation, and standardised pathways, flip lists offer a practical, scalable method to expand elective arthroplasty capacity consistent with GIRFT/HVLC objectives. This model is therefore a viable option for centres seeking to reduce backlogs and improve patient flow while maintaining quality of care.
